# Opportunities to prevent and manage undernutrition to amplify efforts to end TB

**DOI:** 10.5588/ijtld.21.0488

**Published:** 2022-01-01

**Authors:** I. Darnton-Hill, P. P. Mandal, A. de Silva, V. Bhatia, M. Sharma

**Affiliations:** 1 The Boden Collaboration on Obesity, Nutrition, Exercise and Eating Disorders, University of Sydney, Sydney, NSW, Australia; 2 Tuberculosis Unit, , World Health Organization Regional Office for South-East Asia, New Delhi, India; 3 Nutrition and Health for Development Unit, World Health Organization Regional Office for South-East Asia, New Delhi, India

**Keywords:** nutrition policy, infectious disease medicine, public health

## Abstract

The bidirectional relationship between TB and nutrition is well recognized – primary undernutrition is a risk factor for developing TB disease, while TB results in wasting. Although nutrition support is acknowledged as an important intervention in TB programmes, it is seldom afforded commensurate priority for action. TB incidence and deaths worldwide are falling too slowly to meet WHO End TB Strategy milestones, and the number of undernourished people is increasing, likely to be further exacerbated by the ongoing COVID-19 pandemic. Undernutrition needs to be more urgently and intensively addressed. This is especially true for the WHO South-East Asia Region, where the high rates of undernutrition are a key driver of the TB epidemic. The evidence base has been sufficiently robust for clear and workable programmatic guidance to be formulated on assessment, counselling and interventions for TB patients. Many high-burden countries have developed policies addressing TB and nutrition. Gaps in research to date have frustrated the development of more refined programmatic approaches related to addressing TB and malnutrition. Future research can be shaped to inform targeted, actionable policies and programmes delivering dual benefits in terms of undernutrition and TB. There are clear opportunities for policy-makers to amplify efforts to end TB by addressing undernutrition.

## Abstract (fr)

La relation bidirectionnelle entre la TB et la nutrition est bien connue. La sous-alimentation primaire est un facteur de risque de TB active, alors que la TB entraîne une émaciation. Bien que l’appui nutritionnel soit reconnu comme une intervention importante des programmes de lutte contre la TB, elle ne bénéficie que rarement d’une priorité adéquate dans les actions menées. À l’échelle internationale, l’incidence de la TB et les décès associés ne diminuent que trop lentement pour permettre d’atteindre les jalons de la Stratégie de l’OMS pour mettre fin à la TB, et le nombre de personnes sous-alimentées est en hausse et sera probablement exacerbé par la pandémie de COVID-19 actuelle. La sous-alimentation doit être abordée de manière plus urgente et intensive. Ceci est d’autant plus vrai pour la Région OMS d’Asie du Sud-Est, où les taux de sous-alimentation élevés sont un facteur clé de l’épidémie de TB. Les données probantes sont suffisamment solides pour permettre de développer des recommandations programmatiques claires et réalisables en matière d’évaluation, de conseils et d’interventions pour les patients atteints de TB. De nombreux pays à forte prévalence ont élaboré des politiques ciblant la TB et la nutrition. À ce jour, les lacunes de la recherche ont contrarié le développement d’approches programmatiques plus sophistiquées pour lutter contre la TB et la malnutrition. Les études futures peuvent être façonnées de sorte à éclairer des politiques concrètes et ciblées, ainsi que des programmes apportant un double bénéfice en termes de sous-alimentation et de TB. Les responsables politiques peuvent réellement accroître leurs efforts pour mettre fin à la TB en s’attaquant à la sousalimentation.

Global mortality from TB, the leading cause of infectious disease worldwide, has reduced significantly – but slowly. During 2015–2019, the estimated reduction was only 14% against a target of 35% by 2020.^1^ TB’s position as the leading cause of infectious disease worldwide was likely surpassed by COVID-19 in 2020, both in terms of the number of cases and deaths.^2^ The COVID-19 pandemic threatens not only to slow progress further but also to reverse earlier gains. Disruptions to TB services caused by COVID-19 may result in an estimated 0.2–0.4 million additional deaths in 2020, and the economic impact of the pandemic is predicted to worsen at least two key determinants of TB incidence: gross domestic product per capita and undernutrition.^1^

Although the negative effects of TB on the body weight of patients and the positive effects of a good diet are known, inadequate efforts have dogged the nutritional management of TB.^3^,^4^ Undernourished people are three times more likely to develop active TB than their well-nourished counterparts, and an estimated 2.2 million new TB cases globally each year are attributable to undernutrition.^1^

A world free of TB is a common aim of the WHO End TB Strategy and the UN Agenda for Sustainable Development.^3^,^4^ In 2018, the first ever UN General Assembly High-Level Meeting on Tuberculosis adopted a political declaration reaffirming the commitment to end the TB epidemic globally by 2030.^5^ If these targets are to be met, programmatic interventions must be accelerated and expanded, including scaling up actions on addressing undernutrition. While national strategies and TB programmes acknowledge undernutrition as an important risk factor, it seldom receives commensurate prioritisation.

This mini review assesses current evidence, guidance, programmatic experiences and research gaps with regard to TB and nutrition with the aim of promoting renewed attention to mitigate the bidirectional relationship between TB and nutrition While the focus of this paper is the WHO South-East Asia Region, the opportunities outlined could be applied to other settings where undernutrition and TB cooccur.

Ethics review and patient consent for publication was not required as the review was based on secondary data sources.

## TUBERCULOSIS AND UNDERNUTRITION IN SOUTH-EAST ASIA

The WHO South-East Asia Region is home to 26% of the world’s population, yet accounted for 44% of the global TB incidence in 2019 (Table 1) and half of global deaths due to the disease.^1^ Considering the links between nutrition status and active TB, the high prevalence of underweight in Asia is of significant risk (Table 2).^6^

**Table 1:** Estimated TB incidence in countries of the WHO South-East Asia Region, 2019 (Source: WHO Global tuberculosis report, 2020)^1^

Country	Total TB incidence* Mean (range)	Rate/100 000 population (range)*
Bangladesh	361,000 (262,000–474,000)	221 (161–291)
Bhutan	1,300 (960–1,600)	165 (126–208)
Democratic Republic of Korea	132,000 (115,000–150,000)	513 (446–584)
India	2,640,000 (1,800,000–3,630,000)	193 (132–266)
Indonesia	845,000 (770,000–923,000)	312 (285–341)
Maldives	190 (150–240)	36 (28–46)
Myanmar	174,000 (114,000–245,000)	322 (212–454)
Nepal	68,000 (40,000–103,000)	238 (141–359)
Sri Lanka	14,000 (10,000–18,000)	64 (47–83)
Thailand	105,000 (79,000–133,000)	150 (114–191)
Timor-Leste	6,400 (4,200–9,200)	498 (322–711)

* Ranges represent uncertainty intervals.

**Table 2:** Crude estimate of TB prevalence (%) of the underweight * in adults aged ≥18 years (source: Global Health Observatory)^6^

WHO Region	Both sexes % (range)	Male % (range)	Female % (range)
Global	8.9 (8.1–9.9)	8.5 (7.2–9.9)	9.4 (8.1–10.7)
Africa	11.1 (9.6–12.7)	12.2 (9.8–14.8)	9.9 (8.2–11.9)
Americas	1.7 (1.4–2.1)	1.2 (0.9–1.6)	2.2 (1.7–2.8)
Southeast Asia	20.3 (17.6–23.2)	19.8 (15.9–23.9)	20.8 (17.0–24.8)

* Underweight defined as BMI <18 kg/m^2^. Data are for 2016 (latest available).

### Undernutrition as a key driver of TB

Undernutrition is the most common cause of secondary immunodeficiency and affects both innate and adaptive immunity.^7^,^8^ Undernutrition is a risk factor for TB, while TB results in secondary wasting due to increased metabolic demands and anorexia.^9^,^10^ The risk of progression to TB disease is highest soon after infection and is dependent on host risk factors. Although HIV infection is the strongest individual risk factor, undernutrition accounts for a higher proportion of global cases.^4^

Undernutrition is the most common cause of secondary immunodeficiency worldwide, and which affects both innate and adaptive immunity.^7^,^8^ New cases of TB are 12 times higher in people with low body mass index (BMI; <18.5 kg/m^2^);^11^ identified risks increased in TB by undernutrition, include more severe disease, death, delayed sputum conversion, drug-induced hepatotoxicity, malabsorption of anti-TB drugs and relapse after cure.^7^

In 2019, the estimated numbers of cases attributable to undernutrition were 2.2 million globally and close to a million in the South-East Asia Region.^1^ In India, over half of the annual incidence of TB of over a million new cases are attributed to undernutrition.^12^,^13^ Modelling suggests that reducing adult undernutrition could reduce new cases by over 70% in vulnerable states across India.^12^ This is also corroborated by global reviews which suggest that nutritional supplementation could reduce TB incidence and mortality.^14^

Undernourished patients with TB have delayed recoveries and higher mortality rates, while their nutritional status usually improves with TB chemotherapy.^14^ A low BMI puts patients at higher risk of more severe disease, delayed sputum conversion, drug-induced hepatotoxicity, malabsorption of anti-TB drugs and relapse after cure.^12^ Undernutrition, exacerbated due to the COVID-19 pandemic, is likely to fuel the TB epidemic further, specifically in high TB burden countries such as India.^15^

### Implications for formulating policies and programmes to address the malnutrition-TB nexus

National policies on nutrition support in TB are increasingly available; however, strategic and operational planning and implementation are not optimised.^16^ In addition, weaknesses in the evidence base make strategic decisions difficult.

Supplementary feeding programmes are an important approach for nutritionally at-risk populations, but are expensive and complicated to deliver. A Cochrane review of 2018 reported moderate certainty benefits regarding community-based supplementation for food insecure, vulnerable and malnourished populations of all ages, including those with HIV, TB or both.^16^ The ongoing RATIONS (Reducing Activation of Tuberculosis by Improvement of Nutritional Status) Study, a cluster randomised trial of food rations to reduce TB incidence in household contacts of patients with confirmed pulmonary TB in communities with a high prevalence of undernutrition in India is likely to provide further evidence.^17^

When incorporating nutrition care and support for at-risk populations, identifying opportunities for programme delivery and coordination through other health programmes such as childhood immunisation, maternal and child services and HIV programmes should optimise costs and manpower.

### Technical considerations for nutrition therapy in national TB plans

Although the larger role for nutrition is probably in reducing TB incidence, there is also a critical role in treatment. A 2011 Cochrane review, further updated in 2016, assessed any oral nutritional supplement given for at least 4 weeks vs. no nutritional intervention, placebo or dietary advice alone for people being treated for active TB. It reported moderate quality evidence that the provision of free food or high-energy nutritional products probably produces a modest weight gain during treatment.^18^,^19^ A review of 10 studies involving food assistance to vulnerable people with either TB or HIV infection, reported a positive impact in terms of adherence to clinic appointments, adherence to treatment and/or treatment completion in eight studies.^20^

The WHO 2013 guidance on *Nutritional care and support for patients with tuberculosis*, was informed in part by the 2011 Cochrane review and made strong recommendations on assessing nutritional status of TB patients, appropriate counselling, and specifically addressing severe malnutrition.^21^ In 2019, the WHO published *Essential nutrition actions: mainstreaming nutrition through the life-course* which re-emphasised the recommendations of 2013.^22^

The cornerstone of the guidance is that all individuals with active TB should receive 1) an assessment of their nutritional status, and 2) appropriate counselling based on their nutritional status at diagnosis and throughout treatment. Specific nutrition recommendations, including for children and pregnant women, for individuals with active TB and moderate and severe undernutrition, are essentially the same as for those who are undernourished but without TB (Table 3).^14^ Conclusions from other reviews of clinical trials do not conflict with the WHO recommendations.^14^

**Table 3:** WHO recommendations on nutritional evaluation and support in the treatment of TB (source: adapted from Koethe JR, von Reyn CF)^14^

	Recommendations and considerations
Evaluation at baseline	Measure weight, height and calculate BMI
	Counsel all patients regarding an adequate diet with essential macronutrients and micronutrients
	Assess diet if BMI <18.5 kg/m^2^ (marker for increased rate of relapse and mortality)
Nutritional intervention	
All patients	Provide macronutrients if access or adherence to treatment are predicted to be suboptimal (15–30% protein, 25–35% fat, 45–65% carbohydrate)
	If macronutrients are recommended but unavailable, provide a micronutrient supplement at 1 x recommended intake
BMI <16 kg/m^2^ (severe)	Assess for non-dietary causes of malnutrition (HIV, diabetes)
	Provide supplements according to WHO guidelines for severe acute malnutrition
BMI <16–16.9 kg/m^2^ (moderate)	Provide supplements in the outpatient setting until BMI is normalised - intervene earlier with supplement if subject is losing weight during treatment
Multidrug-resistant TB	If BMI ,16.9 kg/m^2^, provide with locally available nutrient-rich foods or supplements; the longer duration of treatment increases risk of undernutrition
Evaluation at 2 months	Assess weight gain; if BMI not yet normal, assess for adherence and comorbid conditions

BMI = body mass index.

### Implications for future research

The overall quantity and quality of research to date is suboptimal but sufficient for strong programmatic recommendations.^22^,^23^ There is an urgent need for fresh research approaches, including well-designed and adequately powered trials focusing on programmatically relevant outcomes and robust evaluations embedded into existing and planned programmes, especially to understand the optimum composition of nutrients. Further research is also needed to understand the pathogenic role of the immune response in wasting, micronutrient and protein-energy deficiency as risk factors for TB, nutrient–gene interactions in vulnerable groups, nutritional support as an affordable adjunctive treatment in resource-poor settings and the role of nutritional support in the management of multidrug-resistant TB (MDR-TB).^3^

Programming evidence from studies that achieve robust long-term adherence and high retention rates, including how they have linked the health system and community services is also essential.^20^

### The need for multisectoral policies and programmes

Effective action on TB and undernutrition requires administratively challenging cross-linkages among a range of sectors, including programmes on TB control, maternal and child health, and immunisation and other sectors such as social security.^21^ Multisectoral actions in public health are frequently called for but are challenging to design and implement; nonetheless, the WHO has provided useful tools to aid the process.

The WHO’s *People-centred framework for tuberculosis programme planning and prioritization* facilitates a systematic approach to country-led, data-driven and people-centred planning, prioritisation and decision-making.^23^ Data sources include nutrition surveys, adherence studies, patient-pathway analyses, national TB prevalence surveys, demographic health surveys, surveys of patient costs, health expenditure surveys and data on economics and poverty.^23^,^24^

The WHO *Multisectoral Accountability Framework for TB* (MAF-TB) can be used to support policy implementation.^25^ MAF-TB supports the process of strengthening accountability through defining stakeholders and their respective roles. The four framework components – commitments, actions, monitoring and reporting, and review – form a cycle for strengthening accountability. Additional annexes help further clarify the defined roles and activities of government, engagement with civil society and affected communities and action on the commitments of the UN General Assembly declaration.^4^,^25^

A useful example of strategic planning and multisectoral programming comes from Timor-Leste, which has a very high burden of TB, as well as malnutrition. A 2016–2017 survey found that 83% of TB patients experienced catastrophic expenditure related to their diagnosis and care – a substantial proportion of which was spent on nutritional supplementation.^26^ This information influenced Timor-Leste’s national strategic plan for ending TB, 2020–2024.^27^ The strategy supports collaboration between the national TB programme and services for reproductive, maternal, new-born, and child health (RMNCH) to improve community detection of TB and optimise contact opportunities and human resources. Routine screening of undernourished individuals will be done both at health facilities and during outreach, and those with TB will be referred immediately and tracked. In addition, all patients with MDR-TB will receive a nutritional support package throughout the full course of treatment to improve adherence.^27^

Similarly, India’s National Strategic Plan for Tuberculosis Elimination, 2017–2025 promotes the operationalisation of actions such as establishing linkage with RMNCH programmes for symptom screening, physical examination and investigations for ruling out TB during child health visits and ante natal/post-natal check-ups.^28^,^29^ It also recommends prioritised support for access to transportation, nutrition, counselling and social welfare, and linking all TB and HIV patients for nutritional support through the public distribution systems. A meticulous evaluation of these initiatives will be vital to inform replication elsewhere. Countries will also need to invest more effort in capacity building and training of health workers in nutrition assessment and counselling.

### Applicability of the South-East Asia experience to the wider global context

Political momentum continues to build around TB, but the WHO Global TB Report 2020 TB attributes inadequate prevention as a key reason for the slow progress.^1^ Addressing TB in in the post COVID-19 context, with its resulting economic downturn, will create further challenges. Since undernutrition is the most significant risk factor, calling up direct and indirect nutritional prevention policies both for the general population and for people living with TB, as being promoted through policy measures in South-East Asia is crucial for global TB elimination efforts. Innovative actions on addressing TB and undernutrition requires resources and investments. Implementation of the recent UN Global Action Plan on child wasting, for which some countries are developing national roadmaps, for example, would support reduction of TB incidence.^30^ Some of the large trials being undertaken in India are likely to inform some of the major gaps in research on science and programming. The experiences of South-East Asia in implementing social protection strategies such as cash transfers, microcredit, and training to improve prevention and mitigation of undernutrition and TB should also prove to be valuable in informing the design, implementation and monitoring of actions in the wider global context.^31^,^32^ Cash transfers, as started under the *Nikhay Poshan Yojna* in India and microcredit initiatives in some other countries are some initial steps towards addressing nutrition issues among TB patients and their families. Some countries also provide food baskets, but these are mostly project-based and through NGO-run projects.

## CONCLUSION

Undernutrition and TB have been described as “strongly linked but ignored”.^33^ This link can no longer be disregarded, as the control of TB in low-resource, food-insecure settings will depend in part on improving nutrition. The quality and quantity of evidence for the role of nutrition in TB prevention and treatment is lower than desirable, with many long-standing research gaps. The world’s undernourished cannot wait for research to be completed. While nutritional support programmes are being implemented, it is imperative to undertake research in new and innovative approaches for scaling up effective and efficient interventions. These, together with robustly assessed programmatic experience and operational and implementation analyses, will prove invaluable to identifying more opportunities to amplify efforts to end tuberculosis through addressing undernutrition.
